# Psychological and Situational Factors Affecting Dropout from Regular Visits in Diabetes Practice: The Japan Diabetes Outcome Intervention Trial-2 Large Scale Trial 004 (J-DOIT2-LT004)

**DOI:** 10.31662/jmaj.2022-0065

**Published:** 2022-08-26

**Authors:** Yusuke Kabeya, Atsushi Goto, Yasuaki Hayashino, Hikari Suzuki, Toshi A Furukawa, Katsuya Yamazaki, Kazuo Izumi, Mitsuhiko Noda

**Affiliations:** 1 Sowa Clinic, Sagamihara, Japan; 2 Department of Health Data Science, Graduate School of Data Science, Yokohama City University, Yokohama, Japan; 3 Department of Endocrinology, Tenri Hospital, Tenri, Japan; 4 Japan Community Health Care Organization Takaoka Fushiki Hospital, Takaoka, Japan; 5 Department of Health Promotion and Human Behavior; Department of Clinical Epidemiology, Graduate School of Medicine and School of Public Health, Kyoto University, Kyoto, Japan; 6 Kawai Clinic, Tsukuba, Japan; 7 Center for Clinical Sciences, National Center for Global Health and Medicine, Tokyo, Japan; 8 Department of Diabetes, Metabolism and Endocrinology, Ichikawa Hospital, International University of Health and Welfare, Ichikawa, Japan

**Keywords:** Dropout from regular visits, Type 2 diabetes, Japan Diabetes Outcome Intervention Trial 2 Large-scale Trial

## Abstract

**Introduction:**

This study explored the psychological and situational factors affecting dropout from regular visits to diabetes care using data obtained from the Japan Diabetes Outcome Intervention Trial 2 (J-DOIT2) Large-scale Trial (LT).

**Methods:**

A total of 2,031 patients with type 2 diabetes who participated in the J-DOIT2-LT were included in the analysis. Responses to a baseline questionnaire with 17 items asking about the experience of dropout from regular visits in diabetes care and its reasons were analyzed using principal component analysis, and factors related to dropout were extracted. Using Cox regression analysis, the association of these factors with the incidence of dropout was investigated.

**Results:**

The mean age of the 2,031 patients was 56.4 ± 5.9 years and 742 (36.5%) were women. They were followed for a median of 392 days, and 125 patients dropped out from regular visits during the follow-up period. In the principal component analysis of the questionnaire, there were four latent factors with eigenvalues of >1.0, which were labeled as “negative perceptions for regular visits,” “social pressure,” “lack of perceived necessity,” and “environmental obstacles” based on the retained items. The Cox regression analysis demonstrated that patients with high scores of “lack of perceived necessity” and “environmental obstacles” had a significantly increased risk of dropout from regular visits.

**Conclusions:**

The present study revealed psychological and situational factors related to dropout, which may be useful for detecting patients at high risk of dropout. Effective measures focusing on such patients to prevent dropouts should be investigated in future studies (The trial registration number: UMIN000002186, registered at the University Hospital Medical Information Network-Clinical Trials Registry).

## Introduction

A significant number of patients dropout from regular visits to diabetes care ^(1), (2)^, resulting in exacerbation of glycemic control and progression of diabetic complications. Preventing patients from discontinuing regular visits is a major concern in diabetes care. Furthermore, identifying the reasons for dropout is of particular interest to medical practitioners. Although several studies ^(3), (4), (5), (6), (7), (8)^ have reported various reasons for dropout and proposed high-risk factors for dropout, the association of such factors with the incidence of dropout is not confirmed. In the Japan Diabetes Outcome Intervention Trial 2 (J-DOIT2) Large-scale Trial (LT), a questionnaire exploring the experience of dropout from regular visits and its reasons was administered to the patients at baseline, which provided us an opportunity to explore the psychological and situational factors associated with dropout. In this study, the factors underlying the responses to the questionnaire were identified and whether the identified factors were associated with the incidence of dropout from regular visits during the follow-up of J-DOIT2-LT was examined.

## Materials and Methods

### Study population and study design

Data from the J-DOIT2-LT were used in this study. The details of the J-DOIT2-LT have been reported previously ^(9)^. The J-DOIT2 was one of the three strategic studies on diabetes funded by the Ministry of Health, Labour, and Welfare with a 5-year target of preventing diabetes and associated complications. The J-DOIT2 was a two-armed cluster-randomized intervention study aimed to evaluate the effectiveness of interventions in reducing dropouts from regular visits to primary care physicians for diabetes treatment. The J-DOIT2 comprised two studies: the J-DOIT2 pilot study (PS) and J-DOIT2-LT. To estimate the effect size and required sample size of the J-DOIT2-LT, J-DOIT2-PS was performed prior to J-DOIT2-LT.

A total of 11 municipal-level district medical associations (DMAs) across Japan participated in the J-DOIT2-LT. The participants were recruited from clinics belonging to the DMAs, and registration started in July 2009. The eligibility criteria were as follows: a diagnosis of type 2 diabetes prior to the registration in the study and being aged between 40 and 64 years. The age groups of 40-64 years were selected, in accordance with the aim of this study to focus on generations who tend to actively work and have relatively high diabetes prevalence. The following patients were excluded from the study: those receiving hemodialysis, hospitalized, bedridden, living in nursing homes, blind, with a history of lower limb amputation, with a history of a malignant tumor within the past 5 years, pregnant, potentially pregnant, having two or more medical doctors in charge of diabetes treatment, or having type 1 diabetes. In total, 2,200 eligible participants were included in the study. Written consent was obtained from all patients. They were randomly assigned to intervention and control groups (954 in the intervention group and 1,246 in the control group). During the study period, the patients assigned to the intervention group received reminders for regular medical visits to their primary care physicians and lifestyle advice, whereas those assigned to the control group did not. They were followed up until September 2010. The dropout from regular visits, which was defined as the failure to visit their primary care physician within 2 months of missing predetermined appointments, was ascertained by clinical research coordinators (CRCs) who visited the clinics every month. Of the 2,200 participants, 169 were excluded because of incomplete baseline questionnaires. Finally, 2,031 participants were included in this study. This study was approved by the ethics committee of the Japan Foundation for Promotion of International Medical Research Cooperation (Approval Code: JFPIMRC-2-220127) and registered in the University Hospital Medical Information Network-Clinical Trials Registry (UMIN000002186).

### Data collection

**Figure 1.**
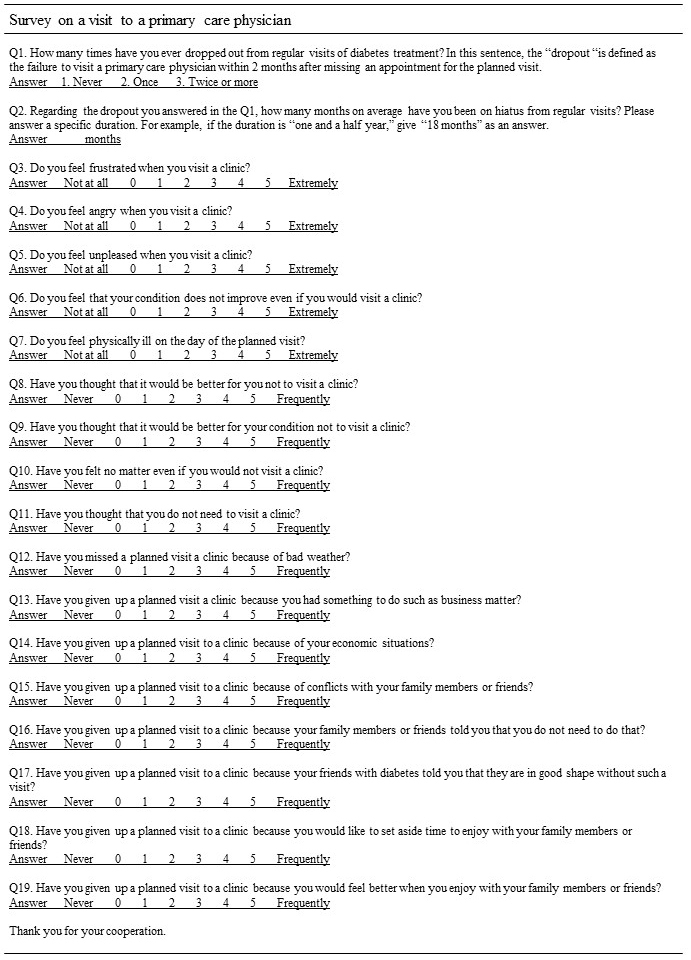
A questionnaire regarding the experience of dropout and its reasons in the J-DOIT2-LT.

Patients’ medical data were collected from medical records by trained CRCs who visited clinics regularly, and the dropout rate was determined. Additionally, a questionnaire was distributed and self-administered at the baseline asking whether a patient had an experience of dropout and the reasons (Figure 1). The questionnaire was developed in reference to the “determinants of relapse” proposed by Marlatt and Gordon ^(10)^. According to the “determinants of relapse,” the possible reasons for dropout were categorized into three groups: intrapersonal factors, environmental factors, and interpersonal factors. The questionnaire consisted of two items (1 and 2) asking about patients’ past experience of dropout from regular visits and the 17 items (3-19) asking about patients’ feelings, thoughts, and experiences of a visit to a primary care physician based on the three factors. Responses to the 17 items were rated on a Likert scale ranging from 0 to 5. In the present study, the data on the patients’ responses to the 17 items were used for the analysis. HbA1c values were originally measured in the Japan Diabetes Society (JDS) units (%). Then, the values were converted to the National Glycohemoglobin Standardization Program (NGSP) units (%) using the following formula (NGSP [%] = 1.02*JDS [%] + 0.25).

### Statistical analysis

#### Exploratory factor analysis

The first step was to establish the latent structure of the responses to the questionnaire using exploratory factor analysis. Initially, the factorability of 17 items was examined. The correlation matrix for the 17 items demonstrated that all 17 items were correlated by at least 0.3 with at least one other item, suggesting that these items were eligible for factor analysis. Second, the Kaiser-Meyer-Olkin measure of sampling adequacy was 0.856, which was above the commonly recommended value of 0.6 ^(11)^. Bartlett’s test of sphericity was significant (χ^2^(136) = 13444.65, *p* < 0.05). A principal component extraction method with varimax rotation was performed. Factors with eigenvalues greater than 1.0 were identified for factor retention. Items with factor loadings greater than 0.6 were retained. For the extracted factors, based on the retained items, a meaningful name was considered.

#### Incidence of dropout from regular visits

Regarding the factors extracted in the analysis, their association with actual dropouts from regular visits was examined. To make the factors practical in a clinical setting, a composite score was created for each factor by averaging the scores of the retained items. The participants were then categorized into three groups according to the composite score so that each group could include an equal number of patients. The frequency of dropouts from regular visits, person-years, and dropout incidence rate were calculated. Hazard ratios (HRs) of dropout from regular visits were also calculated using Cox proportional hazards regression analysis, which was adjusted for age, sex, DMA, and assigned intervention. Regarding the past experience of dropout, no significant interaction was observed in the association between the extracted factors and the incidence of dropout. Consequently, the HRs were calculated without performing stratified analyses. Using the STATA software version 14 (StataCorp, College Station, TX, USA), statistical analyses were conducted. All statistical tests were two-sided, and *p*-values of <0.05 were considered statistically signiﬁcant.

## Results

### Patients’ characteristics

A total of 2,031 patients with diabetes were included in the analysis. Their mean age was 56.4 ± 5.9 years and 742 (36.5%) were women. The mean hemoglobin A1c (HbA1c) level of 1,949 patients whose HbA1c levels were available at baseline was 7.35% ± 1.26%. Table 1 lists the score distributions of the questionnaire. The numbers of patients who have experienced dropout once and twice or more were 189 (9.3%) and 117 (5.8%), respectively. In those who had the experience of dropout, the mean duration of dropout per patient was 14 ± 23 months. Generally, the patients reported relatively low scores for each item. Among intrapersonal factors, item 6 (negative emotion, no improvement) had the highest mean score. Among environmental factors, item 13 (busyness) had the highest score. Regarding interpersonal factors, item 18 (positive emotional state) had the highest score. Nevertheless, the differences in the mean scores between the items in interpersonal factors were minimal.

**Table 1.** Score Distributions of the Questionnaire Asking about an Experience of Dropout from Regular Visits and Reasons in the J-DOIT2-LT.

Item		Components				Score distributions					
						None or no response			Once	Twice or more	
1	Past experience of dropout				n		1,725		189	117	
					(%)		84.9		9.3	5.8	
2	Duration of dropout (months)				Mean 14	(Standard deviation) (23)			Mean 6	(Interquartile range) (3–18)	
Item		Components	Mean	Median		Score distributions					
						0	1	2	3	4	5
3	Intrapersonal	Negative emotion-frustration	0.9	0	n	1,164	398	198	168	61	42
					(%)	57.3	19.6	9.7	8.3	3.0	2.1
4		Negative emotion-anger	0.2	0	n	1,732	181	63	33	10	12
					(%)	85.3	8.9	3.1	1.6	0.5	0.6
5		Negative emotion-discomfort	0.5	0	n	1,424	336	125	92	36	18
					(%)	70.1	16.5	6.2	4.5	1.8	0.9
6		Negative emotion-no improvement	1.0	0	n	1,036	417	208	247	81	42
					(%)	51.0	20.5	10.2	12.2	4.0	2.1
7		Negative physical state	0.4	0	n	1,507	293	129	75	17	10
					(%)	74.2	14.4	6.4	3.7	0.8	0.5
8		Positive emotional state	0.5	0	n	1,542	234	93	102	36	24
					(%)	75.9	11.5	4.6	5.0	1.8	1.2
9		Positive physical state	0.4	0	n	1,646	216	64	68	19	18
					(%)	81.0	10.6	3.2	3.3	0.9	0.9
10		Testing personal control	0.9	0	n	1,130	406	205	206	48	36
					(%)	55.6	20.0	10.1	10.1	2.4	1.8
11		Temptation	0.8	0	n	1,151	445	197	168	37	33
					(%)	56.7	21.9	9.7	8.3	1.8	1.6
									
12	Environmental	Weather	0.4	0	n	1,626	216	67	81	24	17
					(%)	80.1	10.6	3.3	4.0	1.2	0.8
13		Busyness	1.2	1	n	973	417	221	210	110	100
					(%)	47.9	20.5	10.9	10.3	5.4	4.9
14		Economic situation	0.4	0	n	1,670	173	65	58	37	28
				(%)	82.2	8.5	3.2	2.9	1.8	1.4
										
15	Interpersonal	Conflict	0.2	0	n	1,828	126	28	35	10	4
					(%)	90.0	6.2	1.4	1.7	0.5	0.2
16		Social pressure (direct)	0.1	0	n	1,950	56	11	9	3	2
					(%)	96.0	2.8	0.5	0.4	0.1	0.1
17		Social pressure (indirect)	0.1	0	n	1,948	58	12	10	2	1
					(%)	95.9	2.9	0.6	0.5	0.1	0.0
18		Positive emotional state	0.3	0	n	1,684	209	58	61	11	8
					(%)	82.9	10.3	2.9	3.0	0.5	0.4
19		Positive physical state	0.1	0	n	1,849	122	27	28	3	2
					(%)	91.0	6.0	1.3	1.4	0.1	0.1

### Exploratory factor analysis

In the principal component analysis with varimax rotation (Table 2), four latent factors were identified, with eigenvalues of >1.0. These four factors explained 18.6%, 14.4%, 13.8%, and 13.0% of the variance, respectively. Items with factor loadings greater than 0.6 were also identified in each factor. Four items loaded on Factor 1. These four items (3, 4, 5, and 7) were related to intrapersonal negative emotions and negative physical states. This factor was labeled as negative perceptions of regular visits. The two items (16 and 17) that loaded on Factor 2 were related to direct and indirect social pressure, which could be an obstacle to regular visits. This factor was labeled as social pressure. The two items (10 and 11) that loaded on Factor 3 were related to a lack of perceived necessity for regular visits. This factor was labeled as a lack of perceived necessity. The three items (12, 13, and 18) that loaded on Factor 4 identified environmental obstacles to regular visits, such as weather, busyness, and family. This factor was labeled as an environmental obstacle.

**Table 2.** Exploratory Factor Analysis of a Four-Factor Solution Using a Varimax Rotation.

Item		Components	Factor 1	Factor 2	Factor 3	Factor 4
3	Intrapersonal factors	Negative emotion-frustration	0.78	0.03	0.17	0.15
4		Negative emotion-anger	0.69	0.21	0.20	0.06
5		Negative emotion-discomfort	0.83	0.08	0.09	0.13
6		Negative emotion-no improvement	0.57	0.02	0.31	0.17
7		Negative physical state	0.65	0.14	0.03	0.13
8		Positive emotional state	0.48	0.18	0.50	0.02
9		Positive physical state	0.42	0.29	0.48	0.01
10		Testing personal control	0.08	0.06	0.90	0.13
11		Temptation	0.16	0.09	0.87	0.15
					
12	Environmental factors	Weather	0.19	0.19	0.10	0.66
13		Busyness	0.16	0.01	0.22	0.77
14		Economic situation	0.26	0.22	0.13	0.46
					
15	Interpersonal factors	Conflict	0.20	0.60	0.04	0.37
16		Social pressure (direct)	0.09	0.82	0.08	0.05
17		Social pressure (indirect)	0.08	0.83	0.11	0.12
18		Positive emotional state	0.05	0.35	0.13	0.66
19		Positive physical state	0.07	0.56	0.14	0.50
Labeled name			Negative perceptions for regular visits	Social pressure	Lack of perceived necessity	Environmental obstacles
Eigenvalues			5.66	2.01	1.36	1.12
Percentage of total variance			18.6	14.4	13.8	13.0
Number of test measures			4	2	2	3

### Composite score of the identified factors and incidence of dropout

**Figure 2.**
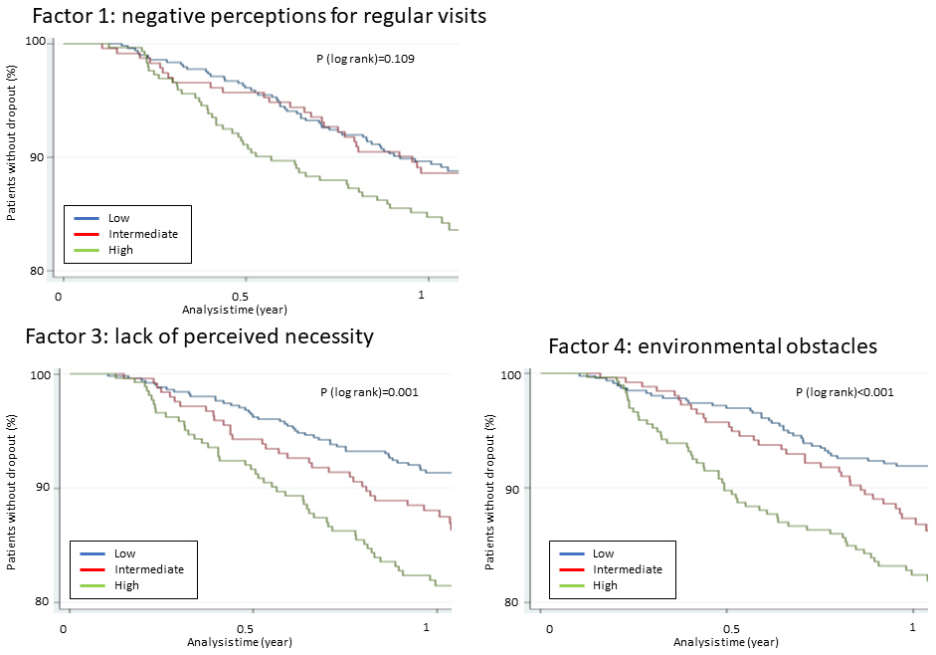
Kaplan-Meier curve estimates of dropout from regular visits by the extracted factors.

Table 3 describes the composite scores of each factor detected in the exploratory factor analysis. Generally, the patients had relatively low composite scores for each factor. Approximately 45%-50% of patients reported a composite score of 0 for each factor, except for Factor 2 (social pressure), in which 95% of patients reported a composite score of 0. Table 4 and Figure 2 present the dropout incidence rates and HRs according to the composite score. Factor 2 (social pressure), in which the distribution of the composite score was highly concentrated at 0, was omitted from the analysis because it seems clinically of no use to categorize patients according to the composite score and estimate the incidence of dropout. During a median follow-up of 392 days (206 days in dropout cases), 125 patients dropped out from regular visits, yielding 2,091 person-years. For each factor, the incidence increased as the composite score increased. When multivariable-adjusted HRs were calculated, statistical significance was observed for Factor 3 (lack of perceived necessity) and Factor 4 (environmental obstacles). As shown in Table 4, patients with high composite scores on Factor 3 (lack of perceived necessity) had a 1.89 (95% confidence interval [CI]: 1.25-2.88) times higher risk of dropout from regular visits when compared with those with low scores. Regarding Factor 4 (environmental obstacles), those with high scores had an increased risk of dropout compared with those with low scores with an HR of 2.03 (95% CI: 1.32-3.12). Further adjustments including the assigned intervention did not change the positive results regarding Factors 3 and 4 as presented in Table 4. No significant interaction by the intervention was observed between the identified factors and dropout incidence (Table 5). Additionally, regarding Factor 3, there might be some effect modification (*p* for interaction 0.059) for men and women because men with its high and intermediate composite scores had a 3.87 (95% CI: 1.56-9.60) and 3.35 (95% CI:1.41-7.98) times higher risk of dropout compared with those with low scores, respectively, whereas women had 1.53 (95% CI: 0.97-2.43) and 0.97 (95% CI: 0.56-1.68) times (data not shown).

**Table 3.** Description of the Extracted Factors and Their Composite Scores Detected in the Explanatory Factor Analysis.

Detected factor	Retained items	Name	Composite score	Mean	Median		Score distributions				
							0	0< to 1	1< to 2	2< to 3	3<
1	3, 4, 5, and 7	Negative perceptions for regular visits	Averaging the scores of Items 3, 4, 5, and 7	0.5	0.25	n	978	705	242	76	30
						(%)	48.2	34.7	11.9	3.7	1.5
2	16 and 17	Social pressure	Averaging the scores of Items 16 and 17	0.1	0	n	1,926	73	17	15	0
						(%)	94.8	3.6	0.8	0.7	0
3	10 and 11	Lack of perceived necessity	Averaging the scores of Items 10 and 11	0.9	0.5	n	1,012	490	271	181	77
						(%)	49.8	24.1	13.3	8.9	3.8
4	12, 13, and 18	Environmental obstacles	Averaging the scores of Items 12, 13, and 18	0.6	0.33	n	926	701	276	88	40
						(%)	45.6	34.5	13.6	4.3	2.0

**Table 4.** Dropout Incidence Rates and Hazard Ratios According to the Extracted Factors.

	The composite score (0–5)		Number of patients	Number of dropouts	Person- years	Incidence rate (per 1,000 person-years)	Crude HR	(95% CI)	Adjusted HR^a^	(95% CI)	Adjusted HR^b^	(95% CI)
	Median	Range										
Factor 1: Negative perceptions for regular visits (Categorical)												
Low	0	0	978	53	1017	52.1	1.00	ref	1.00	ref	1.00	ref
Intermediate	0.25	0.25–0.5	465	26	481	54.0	1.03	(0.65–1.65)	1.03	(0.64–1.66)	0.89	(0.55–1.45)
High	1.25	0.75–4.5	588	46	593	77.6	1.49	(1.00–2.21)	1.46	(0.97–2.18)	1.06	(0.68–1.65)
(Continuous) One-point increase in the composite score							1.23	(1.01–1.50)	1.25	(1.02–1.54)	1.07	(0.85–1.35)
Factor 3: Lack of perceived necessity (Categorical)												
Low	0	0	1,012	45	1053	42.7	1.00	ref	1.00	ref	1.00	ref
Intermediate	1.0	0.5–1.0	490	32	506	63.3	1.4799	(0.94–2.33)	1.38	(0.87–2.18)	1.26	(0.79–2.02)
High	2.0	1.5–5.0	529	48	532	90.2	2.11	(1.41–3.17)	1.89	(1.25–2.88)	1.59	(1.00–2.51)
(Continuous) One-point increase in the composite score							1.30	(1.15–1.48)	1.26	(1.10–1.44)	1.20	(1.04–1.39)
Factor 4: Environmental obstacles (Categorical)												
Low	0	0	926	38	965	39.4	1.00	ref	1.00	ref	1.00	ref
Intermediate	0.33	0.33–0.67	514	35	532	65.8	1.67	(1.05–2.64)	1.57	(0.99–2.50)	1.52	(0.95–2.43)
High	1.67	1.0–5.0	591	52	594	87.5	2.22	(1.46–3.38)	2.03	(1.32–3.12)	1.72	(1.08–2.74)
(Continuous) One-point increase in the composite score							1.31	(1.11–1.56)	1.27	(1.06–1.52)	1.15	(0.94–1.40)

^a^ Adjusted for age, sex, district medical association, and assigned intervention. ^b^Adjusted for age, sex, district medical association, assigned intervention, Factor 1 (categorical), Factor 3 (categorical), and Factor 4 (categorical) except the variable of interest.

**Table 5.** Hazard Ratios of Dropout According to the Extracted Factors, Stratified by the J-DOIT2-LT Intervention.

	Assinged to intervention group				p for interaction
	Yes (n = 872)		No (n = 1,159)		
	Adjusted HR	(95% CI)	Adjusted HR^a^	(95% CI)	
Facor 1: Negative perceptions for regular visits					0.504
Low	1.00	ref	1.00	ref	
Intermediate	1.69	(0.66–4.32)	0.87	(0.50–1.52)	
High	1.73	(0.69–4.36)	1.42	(0.90–2.22)	
Factor 3: Lack of perceived necessity					0.499
Low	1.00	ref	1.00	ref	
Intermediate	1.18	(0.39–3.57)	1.46	(0.88–2.43)	
High	2.71	(1.12–6.60)	1.74	(1.08–2.81)	
Factor 4: Environmental obstacles					0.724
Low	1.00	ref	1.00	ref	
Intermediate	1.32	(0.45–3.87)	1.61	(0.96–2.70)	
High	2.69	(1.08–6.73)	1.93	(1.18–3.14)	

^a^ Adjusted for age, sex, and district medical association.

## Discussion

Using the data obtained from the J-DOIT2-LT, the present study investigated the psychological and situational factors affecting dropouts from regular visits to diabetes care. Risk factors for dropout from regular visits have been proposed in previous studies, including male sex ^(8)^, younger age ^(6), (7), (8)^, no medication for diabetes treatment ^(6), (8)^, high HbA1c values ^(5)^, current smoking ^(4), (5), (8)^, and high alcohol consumption ^(4)^. Nevertheless, psychological and situational factors have not been highlighted. Thus, responses for a questionnaire exploring the experience of dropout from regular visits and its reasons were examined, and the psychological and situational factors at high risk for dropout from regular visits in diabetes care were identified. The association of such factors with the incidence of dropout was examined in the J-DOIT2-LT.

Exploratory factor analysis of the questionnaire suggested a four-factor solution. The derived factors were “negative perceptions for regular visits,” “social pressure,” “lack of perceived necessity,” and “environmental obstacles,” accounting for 59.7% of the variance of the questionnaire responses. When the incidence of dropout was observed according to the composite scores of “negative perceptions for regular visits,” “lack of perceived necessity,” and “environmental obstacles,” it was found that the incidence increased with the composite scores in each factor, although statistical significance was observed only in “lack of perceived necessity” and “environmental obstacles.” Additionally, this study analyzed the effect of a one-point increase in the composite scores of each factor on the risk of dropout by entering the composite scores into the multivariable model as a continuous variable. Consequently, “lack of perceived necessity” was found to be the most influential on the risk of dropout (an HR of one-point increment in the composite score = 1.20 (95% CI: 1.04-1.39)) (Table 4) among the three factors, which could be clinically helpful for considering the approach of patients at high risk for dropout.

Previous studies have reported several reasons for dropouts from regular visits to diabetes care. One study conducted in Japan ^(8)^, which interviewed 152 patients who experienced dropout from regular visits to diabetes care, reported that the most frequent reason for dropout was “busyness with work,” followed by, “considering their diabetes less serious,” “a long distance to a clinic,” “economic situations,” and “busyness with a family matter.” These reasons are consistent with our findings. Busyness with work or family, long distance to the clinic, and economic situations were included in “environmental obstacles,” and “considering their diabetes less serious” was included in “lack of perceived necessity.” Family matters could also be included in “social pressure.” A similar factor was identified in another Japanese study that followed 109 adolescent patients with non-insulin-dependent diabetes and reported that the main reason for dropout from regular visits was a busy schedule. A study in the United States ^(4)^, which analyzed 422 patients with diabetes, also reported that a long distance from home to the clinic was significantly associated with dropout. These findings are consistent with our results.

Although three factors, “environmental obstacles,” “social pressure,” and “lack of perceived necessity” have been discussed in past studies, the reasons related to “negative perceptions of regular visits” have rarely been reported. This seems reasonable because “negative perceptions of regular visits” emerge just before the patients visit a physician and disappear once they skip the visit. Therefore, when the reasons for dropout are interviewed and collected retrospectively, such a reason seems less likely to be reported. “Negative perceptions of regular visits” such as anger or anxiety seem to have more latent reasons. An analysis of the reasons why patients have negative perceptions of regular visits might be useful for finding more essential factors for dropout. At this point, further research is required.

Although this study revealed psychological and situational factors related to dropout from regular visits in diabetes care, whether dropout was preventable in patients with such factors is still unclear. When the analysis was stratified by intervention, no significant effect modification by intervention was observed in the association between the identified factors and the incidence of dropout. The results imply that the intervention conducted in the J-DOIT2-LT might not be helpful in addressing psychological and situational problems, although the intervention could generally motivate patients and reduce dropout. To address such problems, tailored approaches that focus on the four factors found in the present study are suggested. For instance, programs for the management of environmental obstacles or social pressure should be adopted in diabetes patient education. Diabetes educators who are focusing on ameliorating negative perceptions or helping patients understand the importance of regular visits might consider individualized consultations.

**Figure 3.**
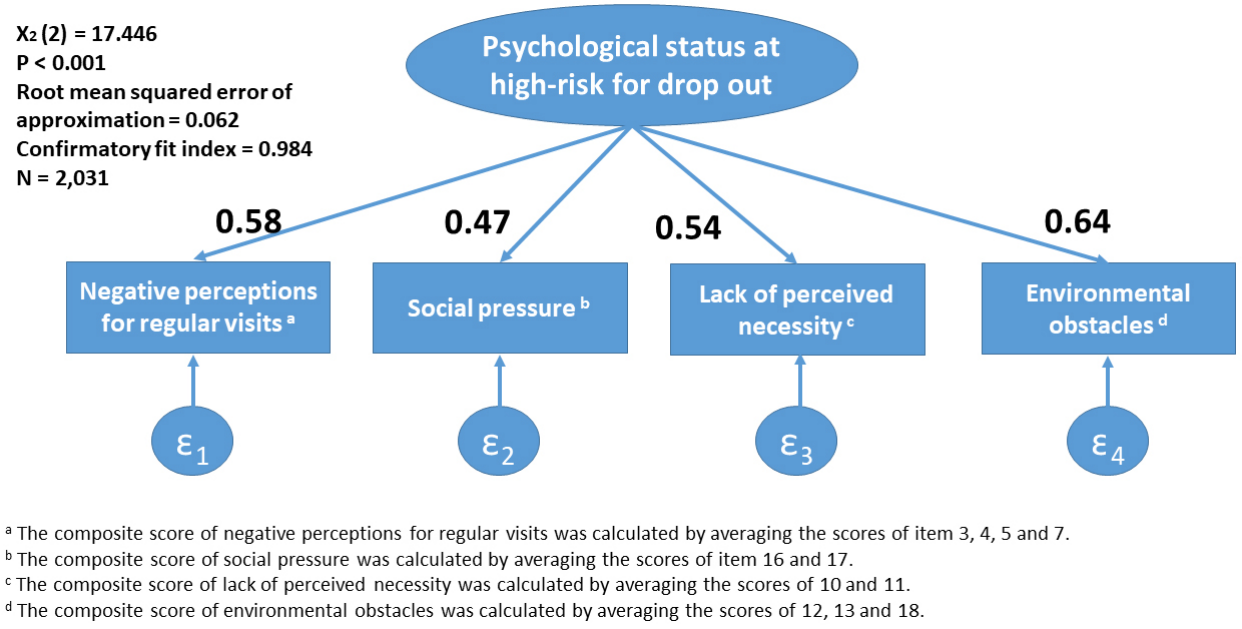
Confirmatory factor analysis with standardized factor loadings explaining the psychological status at high-risk for dropout from regular visits.

The present study has several limitations. First, whether the observed association reflects the causal effect of psychological and situational factors remains unclear. The identified factors, “negative perceptions for regular visits,” “social pressure,” “lack of perceived necessity,” and “environmental obstacles,” could be confounded by other factors such as socioeconomic status or education levels. Nevertheless, the obtained findings on the apparent association might be useful in clinical settings for detecting patients at high risk of dropout. Second, whether the factors detected in the exploratory factor analysis could sufficiently explain the psychological status of individuals at high risk of dropout from regular visits to diabetes care is still unclear. At this point, a simple model explaining the psychological status and situations at high risk for dropout from regular visits was further constructed and a confirmatory factor analysis was performed using the identified factors. The analysis demonstrated a good fit (χ^2^(2) = 17.446, *p* < 0.001, root mean squared error of approximation = 0.062, and confirmatory fit index = 0.984), suggesting that the factors sufficiently explain the psychological status and situations (Figure 3). Third, patients who participated in a randomized controlled trial might be more health-conscious than those who did not. Thus, the sample analyzed in the present study might differ from that in actual clinical practice, which could limit the generalizability of the results. Additionally, this study was performed on Japanese patients via the Japanese healthcare system. Differences in culture or healthcare systems should be considered when these findings are applied to patients in other countries or ethnic groups.

To conclude, the present study explored the psychological and situational factors affecting dropouts from regular visits to diabetes care. The exploratory factor analysis extracted four factors: “negative perceptions of regular visits,” “social pressure,” “lack of perceived necessity,” and “environmental obstacles.” Among the four factors, patients with high scores on “lack of perceived necessity” and “environmental obstacles” had significantly increased risk of dropout than those with low scores on these factors, as to the former, especially among men. By further adjustments, the risk did not change significantly during the intervention conducted in the J-DOIT2-LT, suggesting that the intervention in the J-DOIT2-LT might not be effective for addressing psychological and situational problems. Although our findings might be useful for detecting patients at high risk of dropout from regular visits, no effective ways to prevent patients from facing psychological and situational difficulties from dropout were found. Hence, to reduce the incidence of dropout and improve the quality of diabetes care, further research is warranted to establish effective measures.

## Article Information

### Conflicts of Interest

Yasuaki Hayashino received honoraria from Sumitomo Dainippon Pharma Co., Ltd., and Novo Nordisk.

### Sources of Funding

This work was supported by grants from the Japan Agency for Medical Research and Development (Practical Research Project for Life-Style related Diseases including CVD and Diabetes); the Ministry of Health, Labour and Welfare of Japan (Strategic Outcomes Research Program for Research on Diabetes; Comprehensive Research on Life-Style Related Diseases including CVD and Diabetes H25-016); and a grant from the Japan Diabetes Foundation. The funding source had no role in study design, data collection, data analysis, data interpretation, the writing of the report, or the decision to submit the article for publication.

### Author Contributions

Yusuke Kabeya contributed to the analysis of the results and to the writing of the manuscript. Atsushi Goto, Yasuaki Hayashino, Toshi A Furukawa, and Mitsuhiko Noda contributed to the analysis of the results and the supervision of the findings of this work. Hikari Suzuki, Katsuya Yamazaki, Kazuo Izumi, and Mitsuhiko Noda contributed to the design and implementation of the study. All authors discussed the results and contributed to the final manuscript.

### Approval by Institutional Review Board (IRB)

This study was approved by the ethics committee of the Japan Foundation for the Promotion of International Medical Research Cooperation (Approval Code: JFPIMRC-2-220127) and registered at the University Hospital Medical Information Network-Clinical Trials Registry as UMIN000002186.
